# The *lethal giant larvae* Gene in *Tribolium castaneum*: Molecular Properties and Roles in Larval and Pupal Development as Revealed by RNA Interference

**DOI:** 10.3390/ijms15046880

**Published:** 2014-04-22

**Authors:** Da Xiao, Xiao Liang, Xiwu Gao, Jianxiu Yao, Kun Yan Zhu

**Affiliations:** 1 Department of Entomology, China Agricultural University, Beijing 100193, China; E-Mails: xd@cau.edu.cn (D.X.); gaoxiwu@263.net.cn (X.G.); 2 Department of Entomology, 123 Waters Hall, Kansas State University, Manhattan, KS 66506, USA; E-Mails: liangxiaozju@126.com (X.L.); jianxiu.yao@ag.tamu.edu (J.Y.)

**Keywords:** eclosion, *lethal giant larvae* (*Lgl*), pupation, RNA interference, *Tribolium castaneum*

## Abstract

We identified and characterized the *TcLgl* gene putatively encoding lethal giant larvae (Lgl) protein from the red flour beetle (*Tribolium castaneum*). Analyses of developmental stage and tissue-specific expression patterns revealed that *TcLgl* was constitutively expressed. To examine the role of *TcLgl* in insect development, RNA interference was performed in early (1-day) larvae, late (20-day) larvae, and early (1-day) pupae. The early larvae injected with double-stranded RNA of *TcLgl* (ds*TcLgl*) at 100, 200, and 400 ng/larva failed to pupate, and 100% mortality was achieved within 20 days after the injection or before the pupation. The late larvae injected with ds*TcLgl* at these doses reduced the pupation rates to only 50.3%, 36.0%, and 18.2%, respectively. The un-pupated larvae gradually died after one week, and visually unaffected pupae failed to emerge into adults and died during the pupal stage. Similarly, when early pupae were injected with ds*TcLgl* at these doses, the normal eclosion rates were reduced to only 22.5%, 18.0%, and 11.2%, respectively, on day 7 after the injection, and all the adults with abnormal eclosion died in two days after the eclosion. These results indicate that *TcLgl* plays an essential role in insect development, especially during their metamorphosis.

## 1. Introduction

Cell polarity, which refers to spatial differences in the shape, structure, and function of cells, is fundamental to cellular life and essential for generating cell diversity for all eukaryotic cells [1,2]. It is also essential for various processes including cell division, cell migration, lymphocyte homing and the conduction of nerve impulses [3]. Loss of cell polarity and tissue architecture is the characteristics of malignancy that has severely compromised in a variety of human cancers [4]. Therefore, cell polarity is also considered as a critical parameter in the assessment of tumor grade [5]. To date, three different conserved polarity protein complexes, including the Par, the Crumbs and the Scrib, have been identified. These protein complexes are highly expressed in mammalian epithelial cells [6]. In *Drosophila melanogaster*, the Scrib complex, consisting of Scribble (Scrib), lethal giant larvae (Lgl), and Discs large (Dlg), has also been recognized for its important roles in other forms of polarity, as well as regulation of the actin cytoskeleton, cell signaling, and vesicular trafficking [3].

Lgl is a membrane-associated scaffold that functions in concert with Scrib and Dlg scaffolds, and apical Par6/aPKC complexes to establish apical-basal cell polarity, cell proliferation, differentiation, and tissue organization [2,7–9]. The *Lgl* gene was first discovered in *D. melanogaster* in the 1930s, and identified as a tumor suppressor gene in 1978 [10]. Because the *Lgl* homologs have been identified in various organisms from yeast to humans, this gene has been considered to be evolutionarily conserved among different organisms [7]. Mammals have two *Lgl* homologs, and studies by analyzing the *Lgl1*-knockdown mice have shown the absence of polarity of neural progenitor cells and the development of primitive neuroectodermal tumors *in vivo* [5]. These findings have revealed a critical role of *Lgl1* in the developing brain. The role of *Lgl* in exocytosis has been revealed from the study of *Lgl* homologous genes *Sro7p* and *Sro77p* in yeast that directly interact with Exo84p, a component of the exocyst complex that is essential for targeting vesicles to specific sites of the plasma membrane for exocytosis [2,11].

In *D. melanogaster*, Lgl is known to be associated with cellular membranes and probably involved in cell-cell interactions [12,13]. It may also participate in the formation of the cytoskeletal network, which is necessary for larval development and oogenesis [14,15]. In addition, Lgl is required during embryonic and post-embryonic development to maintain the normal development capacity in *D. melanogaster* [16]. It has also been reported that Lgl participates in the emission of decapentaplegic (Dpp), a member of the transforming growth factor b (TGFb) family, in various developmental processes, including embryogenesis and larval development [17]. In *D. melanogaster*, Lgl is phosphorylated by aPKC (atypical protein kinase C) in the apical region to direct localization of basal components involved in asymmetric cell divisions in neuroblast [18].

Recent studies have shown that Lgl is required for the posterior translocation of oocyte-specific proteins [19] and in regulating differentiation and morphogenetic movement of the ovarian epithelial follicle cells in *D. melanogaster* [20]. *Lgl* is also required for the polarization of embryonic, imaginal disc, and follicular epithelia [4]. Its mutations can result in producing tumors of the brain, the imaginal disc and the follicular epithelium. Lgl is essential for asymmetric cortical localization of all known basal determinants in mitotic neuroblasts [21]. Inactivation of *Lgl* can significantly affect neuroblasts from presumptive optic centers and imaginal disc cells which produce abnormalities in germline, ring gland and salivary glands [10]. Lgl also plays an important role in regulating the asymmetric cell divisions during the development of the peripheral nervous system of *D. melanogaster* [7]. During oogenesis, Lgl plays a critical function at the onset of vitellogenesis and regulates growth of the oocyte, follicle cell migration over the oocyte and their organization in a palisadic epithelium, as well as viability of the germline cells [22]. Recent studies indicate that the presynaptic Lgl scaffold facilitates the assembly of active zone fusion sites to regulate synaptic vesicle cycling [8]. In *Schistosoma japonicum*, Lgl plays an important role in the development of tegument as demonstrated by RNA interference (RNAi) [23].

Studies on different model organisms have demonstrated remarkable conservation of Lgl functions in maintenance of cell polarity and regulation of cell proliferation. However, the exact molecular mechanism by which Lgl functions is still not well understood [9]. To better understand biological functions of *Lgl* in other insect species, we: (1) sequenced and characterized cDNA putatively encoding Lgl from the red flour beetle (*Tribolium castaneum*), an emerging model organism for genetic and genomic studies; (2) examined the developmental stage and tissue-dependent expression profiles of *TcLgl*; (3) investigated the roles of *TcLgl* in insect development by using RNAi. Our results have provided for the first time crucial evidence that *Lgl* is an essential gene during the developmental process of *T. castaneum*. A better understanding of biological functions of *Lgl* may help researchers develop novel RNAi-based pest management strategies by targeting the insect *Lgl* gene.

## 2. Results and Discussion

### 2.1. Analysis of TcLgl cDNA, Deduced Amino Acid, and Genomic Sequences

**Figure 1.**
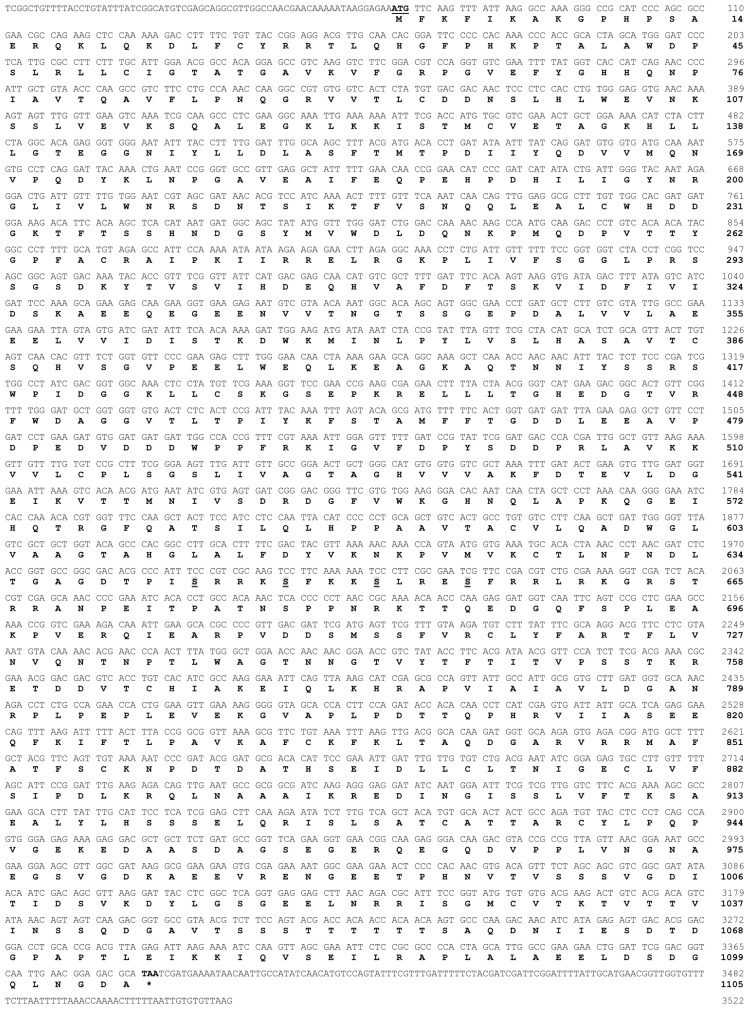
The cDNA and deduced amino acid sequences of *Lgl* gene from *T. castaneum*. The amino acid sequence is numbered from the start of its predicted mature protein. The start codon ATG is bolded and underlined, and the stop codon TAA at the end of the coding region is bolded and marked with an asterisk. The conserved serine phosphorylation sites of the deduced amino acid sequence are bolded and underlined. Both the cDNA and deduced amino acid sequences have been deposited in GenBank (accession numbers: KJ434293).

**Figure 2.**
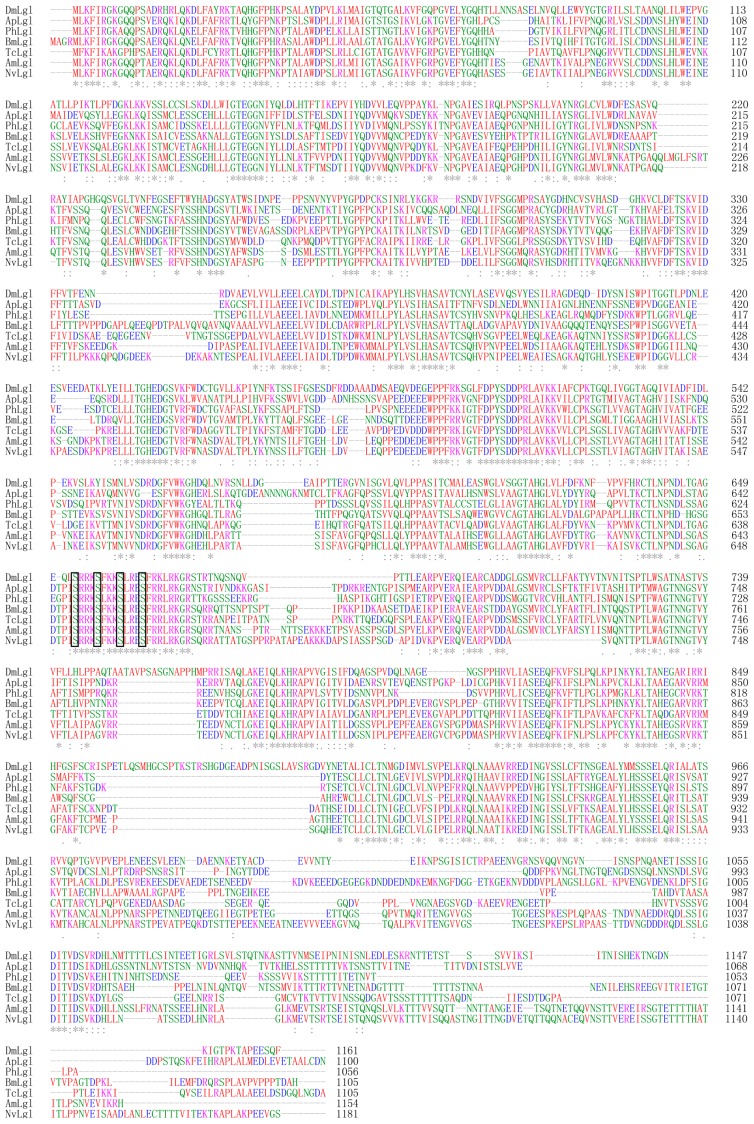
Alignment of deduced Lgl protein sequences from seven representative insect species, including DmLgl (AAG22255.1, *D. melanogaster* Lgl); ApLgl (XP_001948718.2, *A. pisum*); PhLgl (EEB15731.1, *P. humanus corporis*); BmLgl (XP_004921966.1, *B. mori*); TcLgl (KJ434293, *T. castaneum*, this paper); AmLgl (XP_003249432.1, *A. mellifera*); and NvLgl (XP_001604186.2, *N. vitripennis*). Numbering of the amino acid sequences is all started from the *N*-termini of their predicted mature proteins. Identical amino acid residues are indicated by asterisks and conservative substitutions are shown by dots. The conserved serine phosphorylation sites are boxed.

**Figure 3.**
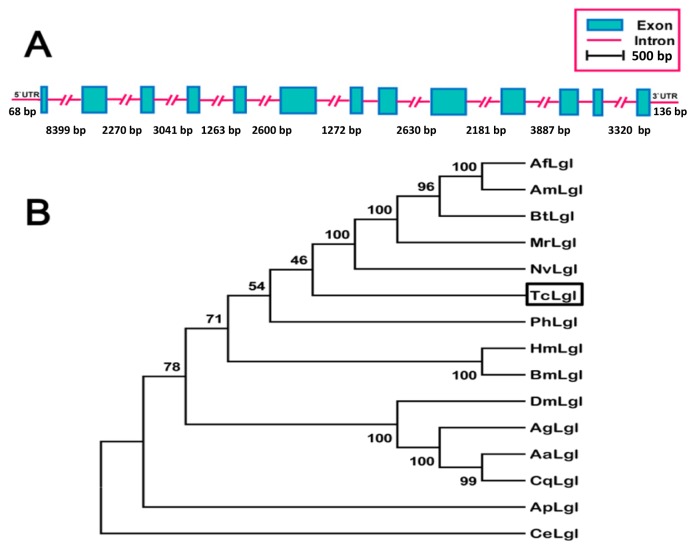
Schematic diagram for the organization of *Lgl* gene from *T. castaneum* (**A**) and the rooted phylogenetic tree of deduced *Lgl* amino acid sequences from the nematode *C. elegans* and fourteen insect species as constructed by the neighbor-jointing method (**B**). All the names used in the tree consist of first letter of the genus, the first letter of the specific name followed by Lgl. Sequences used include: TcLgl (KJ434293, *T. castaneum* this paper); AaLgl (XP_001654112.1, *A. aegypti*); AfLgl (XP_003693724.1, *A. florae*); AgLgl (XP_313781.5, *A. gambiae*); AmLgl (XP_003249432.1, *A. mellifera*); ApLgl (XP_001948718.2, *A. pisum*); BmLgl (XP_004921966.1, *B. mori*); BtLgl (XP_003396710.1, *B. terrestris*); CeLgl (CCD70868.1, *C. elegans*); CqLgl (EDS36704.1, *C. quinquefasciatus*); DmLgl (AAG22255.1, *D. melanogaster*); HsLgl (CBH09297.1, *H. melpomene*); MrLgl (XP_003708503.1, *M. rotundata*); NvLgl (XP_001604186.2, *N. vitripennis*); PhLgl (EEB15731.1, *P. humanus corporis*).

The full-length of *TcLgl* cDNA contains 3522 base pairs (bp), including an open reading frame (ORF) of 3315 bp that encodes a protein of 1105 amino acid residues, and 68- and 136-nucleotide non-coding regions at the 5′- and 3′-ends, respectively (Figure 1). The deduced amino acid sequence from *TcLgl* cDNA exhibits four serine residues located in 643, 647, 651, and 655. *TcLgl* belongs to a typical *Lgl*-type gene, as judged by its sequence similarities with other known insect *Lgls*. All the four sites for serine phosphorylation in Lgl family proteins are conserved (Figure 2). The calculated molecular mass and isoelectric point of the predicted protein are approximately 121.03 kDa and 5.48, respectively. The length of *TcLgl* genomic DNA sequence is 34,603 bp, which contains 13 exons and 12 introns (Figure 3A). The *TcLgl* gene is located on Chromosome 8.

### 2.2. Phylogenetic Relationship of T. castaneum Lgl to Other Insect Lgls

The TcLgl along with other 13 insect Lgls which were retrieved from GenBank formed three distinctive groups representing different orders: Hymenoptera including *Megachile rotundata* (MrLgl, amino acid sequence identity to TcLgl 55.8%), *Apis florae* (AfLgl, 55.4%), *Apis mellifera* (AmLgl, 55.1%), *Bombus terrestris* (BtLgl, 53.8%), and *Nasonia vitripennis* (NvLgl, 53.8%); Lepidoptera including *Bombyx mori* (BmLgl, 53.1%) and *Heliconius melpomene* (HmLgl, 51.1%); and Diptera including *Aedes aegypti* (AaLgl, 43.2%), *Culex quinquefasciatus* (CqLgl, 40.7%), *Anopheles gambiae* (AgLgl, 41.8%), and *D. melanogaster* (DmLgl, 41.6%) (Figure 3B). The remaining two Lgls were from two different orders including Phthiraptera for *Pediculus humanus corporis* (PhLgl, 50.0%) and Hemiptera for *Acyrthosiphon pisum* (ApLgl, 49.7%). Based on the amino acid identity levels, TcLgl appears to be more related to those in Hymenoptera and Lepidoptera than to those in other orders. Its identity level to the nematode (*Caenorhabditis elegans*) Lgl (CeLgl) is only 15.4%.

### 2.3. Developmental Stage- and Tissue-Specific Expression Patterns of TcLgl

**Figure 4.**
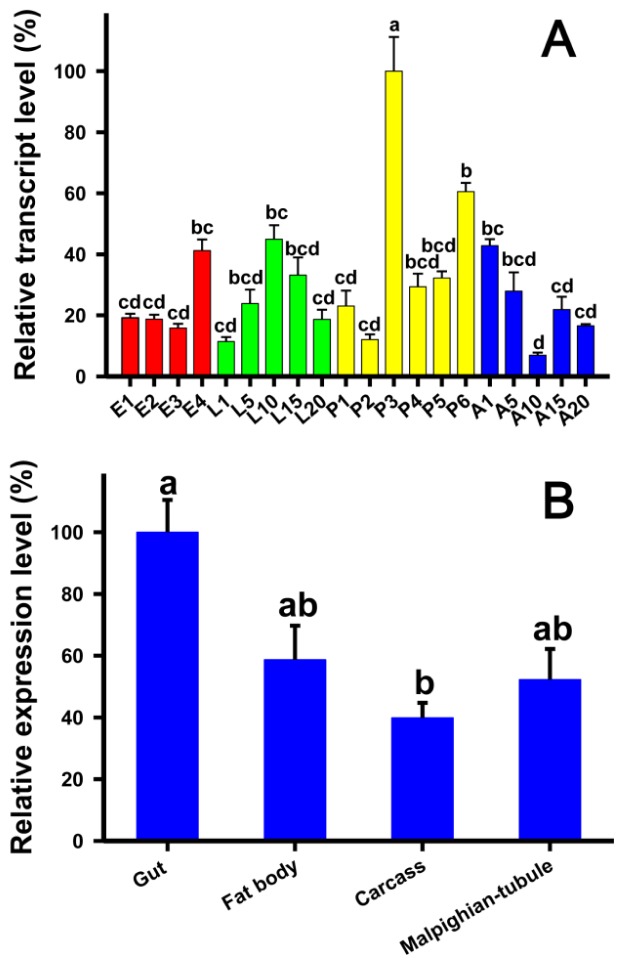
Relative transcript levels of *TcLgl* at different developmental stages (**A**) and in different tissues of 3-day pupae (**B**) of *T. castaneum* as determined by RT-qPCR. E1, E2, E3, and E4 represent 1-, 2-, 3-, and 4-day eggs; L1, L5, L10, L15, and L20 represent 1-, 5-, 10-, 15-, and 20-day larvae; P1, P2, P3, P4, P5, and P6 represent 1-, 2-, 3-, 4-, 5-, and 6-day pupae; and A1, A5, A10, A15, and A20 represent 1-, 5-, 10-, 15-, and 20-day adults, respectively. The carcass represents the remaining body after the brain, ganglia, gut, and fat bodies were removed. For developmental stage/day expression profiling, each sample consisted of 60 eggs, 5 larvae, 5 pupae, or 5 adults. For tissue expression profiling in 3-day pupae, each sample of the guts, fat bodies and carcass was prepared from 30 pupae, and each sample of Malpighian tubules was prepared from 60 pupae. Each developmental stage/day or tissue type was analyzed with three biological samples and each sample was run with two technical replicates. Different letters above the standard error bars indicate significant differences based on ANOVA followed by Tukey’s honestly significant difference (HSD) multiple comparison test (*p* < 0.05). *T. castaneum* ribosomal protein S3 (*TcRps3*) was used as an internal reference gene to normalize the differences among the samples. Relative expression levels for *TcLgl* were calculated based on the highest expressions of *TcLgl* in 3-day pupae (P3) and in the gut as 100% in the developmental stage and the tissue-dependent expression analyses, respectively.

Analyses of the developmental stage-specific expression pattern of *TcLgl* by using reverse transcription quantitative PCR (RT-qPCR) in egg, larval, pupal, and adult stages showed constitutive expression (Figure 4A). The highest expression occurred in the middle pupal stage (3-day pupae), and other expression peaks were found in 4-day eggs and 10-day larvae. The tissues-specific expression pattern in four different tissues, including the gut, fat bodies, carcasses (the remaining body after the brain, ganglia, gut, and fat bodies were removed), and Malpighian tubules, showed the highest expression in the gut although the expression was also moderately high in other tissues examined (Figure 4B).

### 2.4. RNAi of TcLgl in Early (8-Day) Larvae and Its Effect on Survival and Pupation

**Figure 5.**
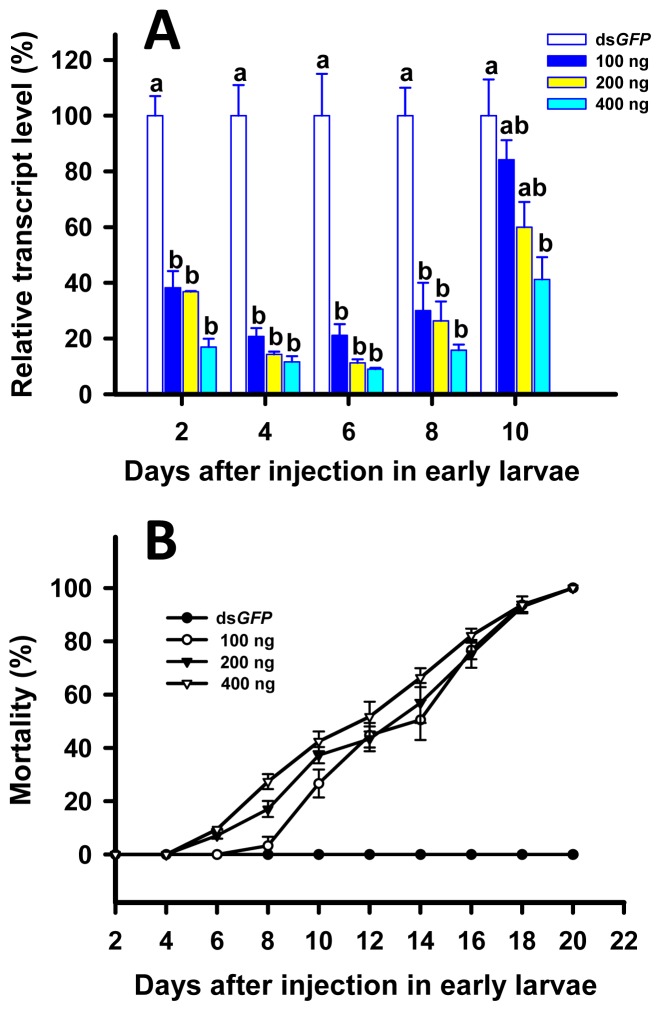
Time-dependent suppression of *TcLgl* transcript in early (8-day) larvae of *T. castaneum* injected with ds*TcLgl* at 100, 200, and 400 ng/larva or ds*GFP* at 400 ng/larva as determined by RT-qPCR (**A**); and the time-dependent larval mortalities in the ds*TcLgl* and ds*GFP*-treated larvae (**B**). The relative expression levels (%) are presented as the mean and standard errors of three replicates; each was performed with a RNA sample prepared from four insects and each sample was run with two technical replicates. The percent mortalities were also determined based three replicates for each dsRNA dose; each replicate with at least 40 early larvae. Different letters above the standard error bars indicate significant differences based on ANOVA followed by Tukey’s HSD multiple comparison test (*p* < 0.05) within the same time point.

We found considerable suppressions of *TcLgl* transcript in all the larvae injected with the double stranded RNA (dsRNA) of *TcLgl* (ds*TcLgl*) at each of the three doses (100, 200, and 400 ng/larva) as compared with the control larvae injected with the dsRNA of *GFP* (ds*GFP*) (400 ng/larva) on days 2, 4, 6, and 8. The *TcLgl* transcript levels were suppressed by 78.9%, 88.7% and 91.0% on day 6 after the injection of ds*TcLgl* at 100, 200, and 400 ng/larva, respectively (Figure 5A). There were no significant differences in suppression of *TcLgl* transcript among the three doses of ds*Tclgl* at days 2, 4, 6, and 8 after the injection. However, the transcript levels bounced back in those injected with ds*TcLgl* at 100 and 200 ng/larva on day 10 after the injection, and the significant difference in the *TcLgl* transcript level was only seen between the ds*TcLgl* and ds*GFP*-injected larvae at the dose of 400 ng/larva. Consequently, the injection of ds*TcLgl* in 8-day larvae led to steadily increasing mortality. By day 20 after the injection, 100% mortalities were observed in ds*Tclgl*-injected larvae at all three doses (Figure 5B). However, the dead larvae did not show any abnormal phenotype.

### 2.5. RNAi of TcLgl in Late (20-Day) Larvae and Its Effect on Survival and Pupation

**Figure 6.**
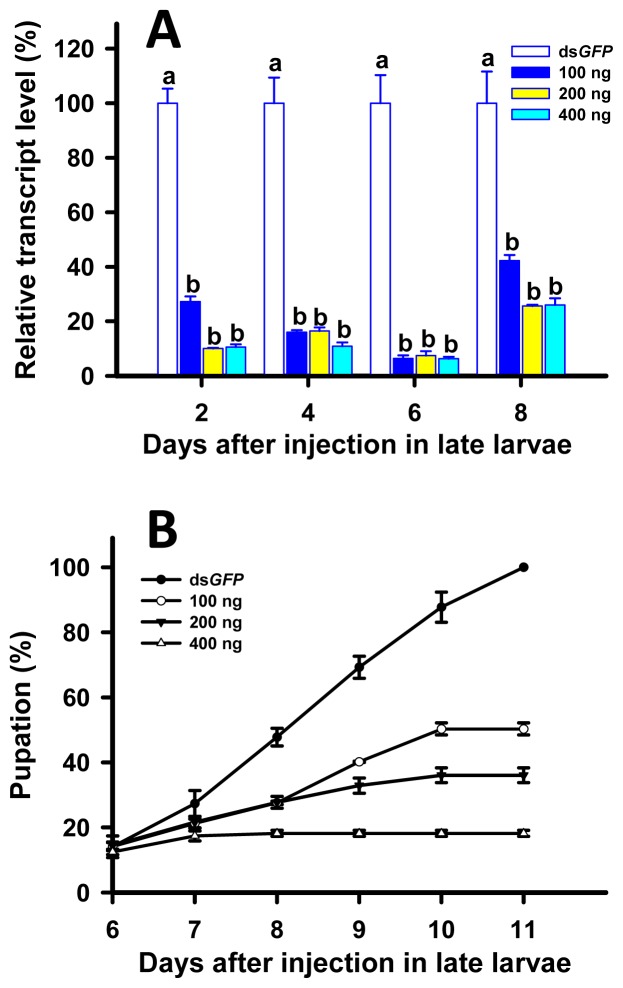
Time-dependent suppression of *TcLgl* transcript in late (20-day) larvae of *T. castaneum* injected with ds*TcLgl* at 100, 200, and 400 ng/larva or ds*GFP* at 400 ng/larva as determined by RT-qPCR (**A**); and the time-dependent pupation rates in the ds*TcLgl* and ds*GFP*-treated larvae (**B**). The relative expression levels (%) are presented as the mean and standard errors of three replicates; each was performed with a RNA sample prepared from four late larvae and each sample was run with two technical replicates. The percent pupations were also determined based three replicates for each dsRNA dose; each replicate with at least 40 late larvae. Different letters above the standard error bars indicate significant differences based on ANOVA followed by Tukey’s HSD multiple comparison test (*p* < 0.05) within the same time point.

The *TcLgl* transcript levels were suppressed by 93.7%, 92.6%, and 93.7% on day 6 after the injection of ds*TcLgl* at 100, 200, and 400 ng/larva, respectively (Figure 6A). However, we did not find significant differences among the three doses of ds*TcLgl* at days 2, 4, 6, and 8 after the injection. On day 8, the transcript levels of *TcLgl* showed some slight recoveries in all the larvae injected with ds*TcLgl* at all the three doses but still showed significant differences from that of the control. As a result, the injection of ds*TcLgl* to 20-day larvae significantly delayed the pupation of the larvae. For the control larvae injected with ds*GFP* at 400 ng/larva, a 100% pupation rate was observed at day 11 after the injection. However, the pupation rates were reduced to only 50.3%, 36.0%, and 18.2% when larvae were injected with ds*TcLgl* at 100, 200, and 400 ng/larva, respectively (Figure 6B). The un-pupated larvae extended their larval stage for more than one week, and then gradually died. Again, these larvae did not show any visible abnormal phenotype. The successfully pupated individuals failed to emerge into adults and died as pupae.

### 2.6. RNAi of TcLgl in Early (1-Day) Pupae and Its Effect on Survival and Eclosion

**Figure 7.**
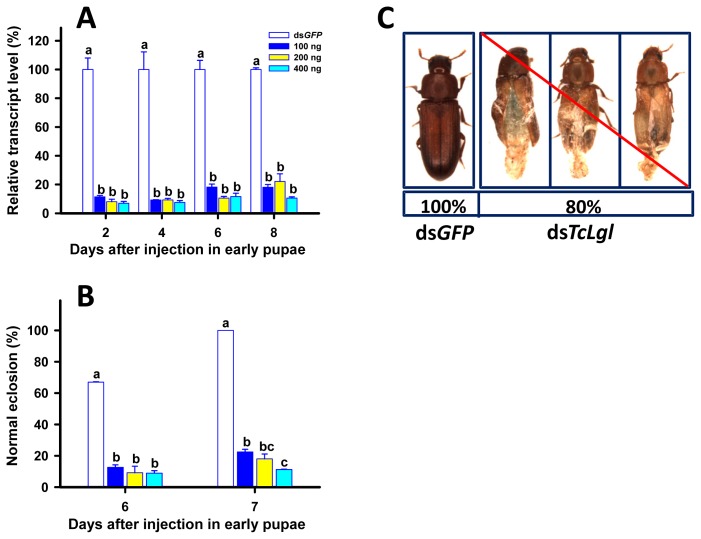
Time-dependent suppression of *TcLgl* transcript in early (1-day) pupae of *T. castaneum* injected with ds*TcLgl* at 100, 200, and 400 ng/pupa or ds*GFP* at 400 ng/pupa as determined by RT-qPCR (**A**); the eclosion rates in the ds*TcLgl* and ds*GFP*-treated pupae (**B**); and the phenotype of abnormal elcosion from the pupae injected with ds*TcLgl* (**C**). The results are presented as the mean and standard errors of three replicates; each was performed with a RNA sample prepared from four early pupae and each sample was run with two technical replicates. The percent normal eclosions were also determined based three replicates for each dsRNA dose; each replicate with at least 40 early pupae. Different letters above the standard error bars indicate significant differences based on ANOVA followed by Tukey’s HSD multiple comparison test (*p* < 0.05) within the same time point.

When early (1-day) pupae were injected with ds*TcLgl* at 100, 200, and 400 ng/pupa, the transcript levels of *TcLgl* were also significantly suppressed on days 2, 4, 6, and 8 after the injection. The *TcLgl* transcript levels were suppressed by 81.9%, 89.4%, and 88.4% on day 6 after the injection of ds*TcLgl* at 100, 200, and 400 ng/larva, respectively (Figure 7A). Such suppressions were highly stable since there were no significant differences in the levels of suppression among all the four time points after the injection. Pupae were highly susceptible to ds*TcLgl*. Only 22.5%, 18.0%, and 11.2% of the pupae emerged to normal adults on day 7 after the early pupae were injected with ds*TcLgl* at 100, 200, and 400 ng/pupa, respectively (Figure 7B). This led to an approximately 80% of an overall abnormal eclosion in the pupae injected with different doses of ds*TcLgl* as compared with normal eclosion in the control pupae injected with ds*GFP* at 400 ng/larva on day 7 after the injection. All the adults with an abnormal eclosion died within two days after the eclosion in ds*TcLgl*-treated insects (Figure 7C).

### 2.7. Discussion

Virtually all the research in insect *Lgl* in the last 80 years has focused on *D. melanogaster Lgl* since the gene was first discovered in the 1930s and later identified as a tumor suppressor gene [10]. In the present study, we identified an *Lgl* homolog from *T. castaneum* (*TcLgl*) and characterized its cDNA and deduced amino acid sequences. Furthermore, we took the advantage of the robust RNAi response of the insect to demonstrate its important biological function in larval development, pupation, and eclosion by using RNAi.

Our results showed that *TcLgl* is a large gene, which encodes 1105 amino acid residues, and is located on Chromosome 8. The domain structure of the Lgl proteins is conserved in eukaryotes and is a hallmark of Lgl family proteins [7]. The deduced protein sequence of *TcLgl* gene exhibits the common features of Lgl family. One of the most important features for Lgls is the presence of four conserved serine phosphorylation sites (S643, S647, S651, and S655 in TcLgl (Figures 1 and 2). These sites are important for serine phosphorylation by atypical protein kinase C (aPKC). Phosphorylation at these residues is important for the function of the protein and its phosphorylation can result in autoinhibitory intramolecular interaction between the Lgl domain and the *N*-terminus [18].

We found that *TcLgl* was constitutively expressed in all developmental stages. The highest expression occurred in the middle pupal stage, and other expression peaks occurred in 4-day eggs and 10-day larvae (Figure 4A). In *D. melanogaster*, however, the amount of Lgl protein reduces towards the end of embryogenesis and remains at a low level during the first and second instars [24]. The protein level then increases again towards the end of the third instar. Thus, an expression pattern of Lgl protein is consistent with the divisions of imaginal disc cells and at least part of the brain cells in the third instar [24]. Indeed, previous analyses have revealed that *Lgl* transcription occurs predominantly during two phases of *D. melanogaster* development including the early embryogenesis and the larval to pupal transition phase [25,26]. However, our results showed that the *TcLgl* transcript maintained at an intermediate level during embryogenesis, rapidly increased before the transition phase from embryo to the first instar larva, peaked during the middle of the larval stage, dramatically increased in the middle of the pupal stage (4-day pupae), and peaked again during the pupal to adult transition phase (Figure 4A).

As the pupal stage showed the highest expression of *TcLgl*, we examined tissue-specific expression pattern of *TcLgl* in the pupal stage. As shown in Figure 4B, *TcLgl* was expressed in all the four tissues examined with relatively high expression level in the gut. Our results are consistent with the expression pattern of Lgl protein in *D. melanogaster* larvae with strong expressions in all imaginal discs, as well as in the proventriculus and midgut epithelium [13].

To reveal the role of *TcLgl* in larval development, pupation and eclosion, we performed detailed functional analyses by using RNAi. Results from our RNAi experiments indicate that *TcLgl* plays an important role in larval development. Specifically, the injection of ds*TcLgl* corresponding to a unique region of *TcLgl* transcript resulted in a dramatic suppression of its transcript level in early larval stage (Figure 5A). Such suppression resulted in 100% larval mortality within 20 days after the injection or before the pupation (Figure 5B). These results suggest that *TcLgl* is essential for larval development and survival. Our results are in agreement with those of a previous study in *D. melanogaster* [27]. That is, the homozygous mutants of *Lgl* alleles were always associated with the tumor formation and the death at the end of larval stage or the beginning of pupal stage.

Our RNAi of *TcLgl* in late (20-day) larvae resulted in two major lines of evidence to support its essential role in pupation. First, the depletion of *TcLgl* transcript by RNAi led to a significantly reduced pupation rate in a dose-dependent manner of ds*TcLgl* (Figure 6B), and the un-pupated larvae eventually died within a short period of time. Second, even if a small proportion of the ds*TcLgl*-injected larvae were able to pupate, these pupae were unable to emerge into adults and died as pupae. These findings are similar to what we found in early (1-day) pupae injected with ds*TcLgl*. The depletion of *TcLgl* transcript appears to primarily block the eclosion process (Figure 7B). For example, only 11.2% of pupae injected with ds*TcLgl* at 400 ng/pupa were able to successfully emerge into adults as compared with a 100% eclosion rate in the control pupae injected with ds*GFP* at 400 ng/pupa on day 7 after the injection. The adults with abnormal eclosion died with the exuviae attached to their bodies in two days after the eclosion (Figure 7C).

Our results are in agreement with those observed in the *Lgl* mutant larvae [28] and the recessive mutants for the *Lgl* gene [29] in *D. melanogaster*. The *Lgl* mutant larvae were not able to undergo the metamorphosis as a consequence of defective function of ring glands [28], which is a compound structure containing the prothoracic glands, corpus allatum, and corpus cardiacum. Furthermore, the recessive mutants for the *Lgl* gene might indirectly influence the synthesis of ecdysone from dietary cholesterol [29]. More recently, another study showed that the imaginal discs and brain of the *Lgl* mutant larvae of *D. melanogaster* overgrew and the resultant giant larvae died without entering metamorphosis [22]. Our results from the RNAi experiments in early larvae (Figure 5), late larvae (Figure 6), and early pupae (Figure 7) are in agreement with those reported in the *Lgl* mutants of *D. melanogaster* and support our notion that Lgl plays an essential role in insect development and metamorphosis. However, further research would be necessary to better explain the underlying molecular causes for the RNAi-induced phenotypic effects in *T. castaneum*. Such studies may help researchers understand the molecular mechanism by which Lgl functions in insects and other organisms.

## 3. Experimental Section

### 3.1. Insect Culture

The Georgia-1 (GA-1) strain of *T. castaneum* was reared on whole-wheat flour containing 5% (by weight) of brewers’ yeast at 30 °C and 65% RH in growth chamber in the Department of Entomology at Kansas State University (Manhattan, KS, USA).

### 3.2. Total RNA Isolation and Reverse Transcription

Total RNA was isolated from *T. castaneum* samples using TRIzol reagent (Invitrogen, Carlsbad, CA, USA) and RNA concentration was measured using NanoDrop 2000 spectrophotometer (Thermo Fisher Scientific, Waltham, MA, USA) at 260 nm. After the total RNA (1.0 μg) was treated with DNase I (Fermentas, Glen Burnie, MD, USA) to remove possible genomic DNA contamination, the first-strand cDNA was synthesized by using First Strand cDNA Synthesis Kit (Fermentas) with oligo (dT)_18_ as the primer in a 20-μL reaction system. The first-strand cDNA was used in following analyses.

### 3.3. Subcloning and Sequencing of cDNA

Four pairs of gene-specific primers were designed based on the *TcLgl* gene prediction in Beetlebase (Accession number: Tc015986) to amplify overlapping fragments by PCR for assembling the full-length cDNA corresponding to the entire protein coding regions (Table 1). The PCR products were subjected to electrophoresis on 1% agarose gel. The PCR bands were excised and purified using QIAEX II Agarose Gel Extraction Kit (Qiagen, Valencia, CA, USA). The purified PCR fragment was ligated into a pCR™2.1 Vector (Invitrogen). The ligation mixtures were then used to transform DH5α bacterial cells. Plasmids were isolated from the bacterial cells and sequenced by DNA Sequencing Facility at Kansas State University (Manhattan).

### 3.4. Analyses of TcLgl cDNA, Deduced Amino Acid, and Genomic Sequences

The amino acid sequence of a putative TcLgl protein was deduced from its cDNA, and molecular mass and isoelectric point of the deduced protein were calculated by using online tools [30]. Multiple amino acid sequence alignment of all known insect Lgls found in GenBank was carried out using ClustalW [31]. The phylogenetic tree of the deduced amino acid sequences of Lgls from *C. elegans* as an outgroup and the insect species available in GenBank was generated using the neighbor-joining algorithm by using MEGA 5.0 [32]. To evaluate the branch strength of the tree, a bootstrap analysis of 1000 replications was performed. The exon/intron organization of *TcLgl* was revealed by comparing the full-length cDNA sequence with its corresponding genomic sequence [33].

### 3.5. Analysis of Expression Profiles by RT-qPCR

The relative transcript levels of *TcLgl* were analyzed by RT-qPCR using SYBR Green by using the Bio-Rad iCycler iQTM multi-color real-time PCR detection system (Bio-Rad Laboratories, Hercules, CA, USA) based on the method of Giulietti, *et al*. [34]. For developmental expression profiling, samples were prepared from embryos (1-, 2-, 3-, and 4-day eggs), larvae (1-, 5-, 10-, 15-, and 20-day), pupae (1-, 2-, 3-, 4-, 5-, and 6-day) and adults (1-, 5-, 10-, 15-, and 20-day). Total RNA was extracted from each stage and tissue sample by using TRIzol reagent (Invitrogen) and 1.0 μg of total RNA was used for cDNA synthesis by using First Strand cDNA Synthesis Kit (Fermentas). The gene-specific primers (Table 1) were designed by using the Beacon Designer 7.0 software (PREMIER Biosoft International, Palo Alto, CA, USA) and *TcRps3* in *T. castaneum* was used as an internal reference gene [35].

The optimized quantitative PCR program consisted of an initial denaturation at 95 °C for 5 min followed by 40 cycles of 95 °C for 15 s, 61.4 °C for 30 s, and 70 °C for 30 s. At the end of the PCR, amplification specificity was verified by obtaining the dissociation curve, in which the samples were cooled to 55 °C after denaturing and then the melting curves were obtained by increasing 0.5 °C/10 s for each cycle with a total of 80 cycles until reaching 95 °C to denature the double-stranded DNA. The specificity of each reaction was evaluated based on the melting temperatures of the PCR products. RT-qPCR was performed with three biological replicates, each with two technical replicates. The transcript levels of *TcLgl* were expressed as normalized transcript abundance using *TcRps3* as an internal reference gene [35]. The relative *TcLgl* transcript levels were calculated according to the 2^−ΔΔ^*^C^*^t^ method [36,37].

### 3.6. Functional Analysis of TcLgl

RNAi was carried out to evaluate the role of *TcLgl* in *T. castaneum* development. dsRNAs were synthesized using MEGAscript^®^ RNAi Kit (Ambion, Austin, TX, USA) according to manufacturer’s manual. Relevant information on the primers used for dsRNA synthesis was shown in Table 1. The early (8-day) larvae, late (20-day) larvae, and early (1-day) pupae were selected for injections of dsRNA of *TcLgl* (ds*TcLgl*) at three doses (100, 200, and 400 ng/larva) or dsRNA of the green fluorescent protein gene (ds*GFP*) as controls. To reduce the number of controls, we chose only one dose (400 ng/larva) for ds*GFP* because our preliminary experiments with different doses of ds*GFP* showed no difference in the control mortality of *T. castaneum* and no effect on the expression of *TcLgl*. The mortality owing to the ds*GFP* injection was less than 10%. The injected insects were reared under standard conditions and phenotype was recorded every day after the injection. RT-qPCR was used to monitor the change of the *TcLgl* transcript level after the injection as described above.

### 3.7. Statistical Analysis

The percent transcript level of *TcLgl* in RT-qPCR analysis from the RNAi experiments were calculated by dividing the relative expression value (REV) in the ds*TcLgl*-injected insects by the REV in the ds*GFP*-injected insects. The percent data, from the developmental stage and tissue-specific expression analyses, and the RNAi experiments, were first transformed using arcsine square root transformation, and the transformed data were subjected to ANOVA followed by Tukey’s HSD multiple comparisons to separate the means among the stages, tissues or dsRNA treatments by using ProStat software (Poly Software International, Pearl River, NY, USA).

## 4. Conclusions

Since the *Lgl* gene was first discovered in *D. melanogaster* approximately 80 years ago, previous research has revealed evolutionally conserved mechanisms of Lgls in regulating cytoskeleton and membrane traffic for the generation and maintenance of cell polarity in different organism systems. However, much of such evidences in insects have been from the studies of the *Lgl* mutants of *D. melanogaster*. In this study, we sequenced and characterized cDNA putatively encoding Lgl from *T. castaneum* and examined the developmental stage and tissue-dependent expression profiles of *TcLgl*. By using RNAi tools, our study provided for the first time crucial evidence that *TcLgl* plays an essential role in insect development and metamorphosis. Our results also suggest that insect *Lgl* gene can potentially serve as an excellent target gene for developing RNAi-based pest management strategies due to its conserved and essential function in insect development.

## Figures and Tables

**Table 1.** Primers used to amplify *TcLgl* cDNA sequences, synthesize dsRNA and analyze transcript levels.

Application of Primers		Sequence (5′-3′)	Tm (°C)	Product Length (bp)
PCR for cNDA sequence	*TcLgl-*1-F	TCGGCTGTTTTACCTGTATTTATC	59.11	875
	*TcLgl-*1-R	GCCATCATTATGTGAGCTTGTG	60.53	
	*TcLgl-*2-F	GTGCCGTTGAGGCTATTTTT	59.23	1036
	*TcLgl-*2-R	CCGGCAACAATCAAACTTC	59.11	
	*TcLgl-*3-F	AGTACAGCGATGTTTTTCACTGG	60.58	1030
	*TcLgl-*3-R	CTGGAAGTGGTGCTACCCC	60.52	
	*TcLgl-*4-F	CTATACCTTCACGATAACGGTTCC	60.15	1220
	*TcLgl-*4-R	CTTAACACACAATTAAAAAGTTTTGGT	58.32	

dsRNA synthesis	ds*GFP*(T7)-F	GGATCCTAATACGACTCACTAT	60.23	305
		AGGGTGACCACCCTGACCTAC		
	ds*GFP*(T7)-R	GGATCCTAATACGACTCACTAT	60.4	
		AGGGTTGATGCCGTTCTTCTGC		
	ds*TcLgl*(T7)-F	TAATACGACTCACTATAGG	60.11	385
		GGACGTTGCAACACGGATTC		
	ds*TcLgl*(T7)-R	TAATACGACTCACTATAGG	60.40	
		GTGTCATCGTAAAGCTTGCCA		

RT-qPCR	*TcLgl*(Q)-F	GACGGATGGCTTTTGCTA	61.6	141
	*TcLgl*(Q)-R	CGGCATTCAACTGTCTCT	61.2	
	*TcRps*3-F	CCGTCGTATTCGTGAATTGACTT	61.1	130
	*TcRps*3-R	TCTAAGAGACTCTGCTTGTGCAATG	61	
